# Translation and Validation of Italian Version of Index of Dental Anxiety and Fear (IDAF-4C+): A Cross-Sectional Study

**DOI:** 10.3390/dj9120149

**Published:** 2021-12-10

**Authors:** Stefano Salgarello, Maria Luisa Garo, Corrado Paganelli, Antonio Vita, Matteo Salvadori, Elisabetta Audino

**Affiliations:** 1 Department of Medical and Surgery Specialties, Radiological Sciences and Public Health, Dental School, University of Brescia, 25123 Brescia, Italy; stefano.salgarello@unibs.it (S.S.); corrado.paganelli@unibs.it (C.P.); salvaz.dori@gmail.com (M.S.); bettaz92@gmail.com (E.A.); 2 Department of Clinical and Experimental Sciences, University of Brescia, 25123 Brescia, Italy; antonio.vita@unibs.it

**Keywords:** dental anxiety, dental fear, IDAF-4C+, validity, reliability, Italian version

## Abstract

Dental anxiety (DA) is defined as unreasonable apprehension toward dental procedures. About 4–20% of the worldwide adult population presents DA, with peaks of 30% in the younger population. Managing patients with dental anxiety and fear with a reliable and valid instrument is necessary to understand the multidimensional dimensions of dental fear. This work aimed to validate the Index of Dental Anxiety and Fear (IDAF-4C+) into Italian. Two hundred and eighty dental students attending an Italian university were enrolled. The IDAF-4C+ was translated by experts and a native English translator, blinded to the original version. The Modified Dental Anxiety Scale (MDAS) was used to assess the validity of IDAF-4C+. Spearman correlation coefficients and Exploratory Factorial Analysis (EFA) were used. Reliability was evaluated by Cronbach’s alpha. The reliability of the Italian version of IDAF-4C+ was good (Cronbach’s alpha = 0.88). Correlation between IDAF-4C+ and MDAS ranged between 0.42 to 0.68. From EFA, one factor explained 58.76% of the common variance. Women showed a higher level of dental fear. The Italian IDAF-4C+ is a valid and reliable tool to assess DA in any clinical context. This instrument allows for a proper understanding and management of DA, and therefore a better patient oral health-related quality of life and compliance with the dentist’s instructions.

## 1. Introduction

Dental anxiety (DA) is defined as unreasonable apprehension toward dental procedures or the whole context of the dental setting [1,2]. Its onset is usually associated with physiological and emotional arousals [3,4,5] and could significantly deteriorate the patients’ quality of life and oral health [6]. Considered one of the most relevant causes of avoidance of dental treatments [7], DA is a substantial public health problem [8,9] that may interfere significantly with the individual’s everyday life [10].

About 36% of the adult population presents with a mild anxiety level for dental treatments [11]. The prevalence of DA ranges between 4% and 20% of the worldwide adult population, with peaks of 30% in the younger population [12,13]. These percentages significantly increase in patients with irreversible pulpitis, when over 80% showed mild to severe dental anxiety [14].

DA is a multidimensional problem composed of two elements: anxiety and fear. The first is a cognitive factor, a future-oriented state responding to an anticipated threat, while the second is a contextual factor, a present-oriented emotional state responding to an immediate threat [15]. In the most severe cases, DA is diagnosed as a dental phobia or “odontophobia”, a disabling or causing extreme distress according to DSM-V (*Diagnostic and Statistics Manual of Mental Disorder, Fifth Edition*) and ICD-10 (*International Classification of Disease, Tenth Revision*) [16,17,18,19].

Given the broad spectrum of severity, DA could be considered as a predictor of patient behaviors [14], a critical factor such as in “over-expectation” of pain [20] during and after dental treatments, and the first and foremost an oral-health problem itself [21].

Many studies have investigated the etiology of dental anxiety in adults and the young, identifying direct and indirect causes [10]. Negative dental experiences [22] and learning through other people’s experiences or the media’s influence, together with an anxious personality trait and environmental factors [14], could determine the onset of DA. Moreover, the possibility of experiencing pain during dental visits might significantly contribute to DA development [23,24]. As described by Berggren et al., (2001), DA is, in many cases, the trigger of a vicious circle [19]: DA leads to the avoidance of dental treatments [7], this behavior causes poor oral health and complications [25,26], which in turn leads to more invasive procedures [12,27] given the higher risk of periodontal disease [25] and a substantial decrease in general well-being and quality of life [3,28].

For the dental team, DA is also a source of stress. Patients with DA require additional time to implement dental care, complain of more discomfort and pain [14], and need specific skills to adopt preventive strategies to minimize the invasiveness of dental treatments [11].

Therefore, assessment of DA is a part of the clinical evaluation of the patient’s condition. Knowing the magnitudes of DA may enhance the patient’s management [29], reduce activities that can induce the patient’s DA, and help clinicians plan an optimal approach in the most invasive procedures [30,31].

Many psychometric scales have been developed to identify patients with DA and assign them a degree of severity. Recently, the Index of Dental Anxiety and Fear (IDAF-4C+) has been considered a valuable scale to assess DA in the adult population, and it has been translated into different languages [27,32,33,34,35] because it offers more information to clinicians and researchers than the other anxiety scales [36]. IDAF-4C+ is strongly based on DSM-IV specific phobia categories, helping researchers and clinicians better determine emotional, behavioral, physiological, and cognitive components of the anxiety and fear response.

According to Censis and ANDI (*Associazione Nazionale Dentisti Italiani*) research, over 17 million Italians have never made even one dental visit in their life. Furthermore, about 80% of Italians declare that they are afraid of the dentist, 20% are even terrified of them, to the point of reaching panic and anxiety attacks [37]. In Italy, several studies investigating the role of dental anxiety and fear in children and adults relied on validated tools, mainly the Modified Dental Anxiety Scales (MDAS), translated and validated by Facco et al. [38]. Such studies demonstrated that third molar extraction and periodontal or implant surgery, the most invasive dental procedures, may quickly generate anxiety among patients.

Nevertheless, a more robust psychometric instrument, designed for adults and able to identify odontophobia, from mild to severe, can help clinicians adopt more personalized techniques to reduce anxiety. Fear of phobia MDAS, although easy and with good internal consistency and test-retest reliability, could not reveal crucial factors in the patients’ approach to dental treatments [39].

To date, there is no validated and reliable translation of IDAF-4C+ into Italian. Beaton et al., (2000) suggested that a cross-cultural adaptation process is necessary to maintain the original instrument content validity across different cultures, adapting language and meaning to the specific cultural context in which the tool will be used [40]. This process ensures the maintenance of psychometric properties such as the validity and reliability of the scale and its items [40].

This cross-sectional study aimed to translate and validate IDAF-4C+ into Italian, testing its reliability and validity in comparison with the Modified Dental Anxiety Scale (MDAS) in a sample of undergraduate students, indicated by previous studies with subjects with a higher level of anxiety than the older population [41], more prone to dental anxiety [42], and vulnerable to oral health negligence [43].

## 2. Materials and Methods

### 2.1. Translation

The original English version of the IDAF-4C+ was translated using a forward- and back-translation procedure [40,44]. First, two research team members who were fluent in English independently translated the original scale into Italian. Then, the phobia module of IDAF-4C+ was translated according to DSM-V criteria for the simple phobia diagnosis and under the supervision of an experienced psychiatrist. A new Italian version of the IDAF-4C+ was subsequently designed based on the original and two translated versions. Later, this new version was submitted to a native English translator, blinded to the original version and fluent in Italian, who translated it again into the original language. Afterward, the research team discussed the discrepancies. Finally, an expert panel on dental fear at the Dental School of the University of Brescia reviewed the final version.

### 2.2. Measures

The Index of Dental Anxiety and Fear (IDAF-4C+) is composed of three independent modules. The first module (IDAF-4C) contains eight items with responses, ranging on a 5-point Likert scale from “disagree” (score = 1) to “strongly agree” (score = 5) measuring both anxiety (i.e., anticipatory anxiety) and fear (i.e., treatment-related anxiety). This core module aims to measure four components of dental anxiety: cognitive, physiological, behavioral, and emotional.

A total score was calculated for each participant by averaging responses to items. An IDAF-4C+ mean score ≤2.5 identified patients without fear or with a moderate fear; an IDAF-4C+ mean score ranging between 2.5 and 3.5 classified patients with moderate to high fear, and IDAF-4C+ mean score ≥3.5 identified patients with high to extreme fear [33,34,35].

The module IDAF-P aims to provide a screening of dental phobia diagnosis according to DSM-V-based criteria. It consists of five items with “yes” or “no” responses: three items to diagnose dental phobia, and the other two to provide a differential diagnosis from social phobia and panic disorder according to the DSM-V [17,33]. Patients who reported IDAF-4C score ≥3 and responded “yes” to the first three items and “no” to the two last items were diagnoses with dental phobia [32,34,45]. To improve the clearness of the two items, we added simple examples of what we meant with “going to the dentist significantly affects my life in some way” and “panic attack”.

The third module, the stimulus module (IDAF-S), composed of ten items with five responses, ranging from “Not at all” (score = 1) to “Very much” (score = 5), aims to determine potentially anxiogenic stimuli present in a dental setting [34]. The English and Italian versions of the validated instrument are reported in the Supplementary Materials.

The Modified Dental Anxiety Scale (MDAS) is a widely used measure of dental fear [46] and was adopted in this study to validate the Italian version of IDAF-4C+. It consists of five items with five possible responses to each question, ranging from “Not anxious” (score = 1) to “Extremely anxious” (score = 5). A total score was calculated for each participant by adding scores of each item [45]. According to Yuan et al., (2008), MDAS total score was categorized into “No fear” (total score: 5–9), “Low/Moderate Fear” (total score: 10–18), and “High fear” (total score: ≥19). MDAS has reported an optimal validity, high internal consistency (Cronbach’s alpha = 0.94), and a strong reliability over-time (ICC = 0.93). Furthermore, thanks to its two-factor structure, MDAS measures anticipatory anxiety (items 1 and 2, range 2–10) and treatment-related anxiety (items 3, 4, and 5, range 3–15) [34,47].

### 2.3. Participants

The final Italian version of the IDAF-4C+ was administered to 280 students attending the University of Brescia. An online questionnaire was administered from 6 to 16 January 2021 through Google Forms. Students were recruited via email and were provided with information on the study. Participation in the questionnaire was voluntary, anonymous, and without any form of remuneration. This study did not fall under human research’s Italian law, and the Ethical Committee did not ask for specific approval.

The first part of the questionnaire was the Italian translation of the IDAF-4C+ composed of 23 items. The second part was the Italian version of MDAS, already validated by Facco et al., (2015) [38]. Finally, the third part consisted of three questions about the participants’ sociodemographic characteristics.

The sample size was determined a priori (population: about 1600 students, confidence level: 90%, margin of error: 5%; minimum sample size required: 233 respondents).

### 2.4. Statistical Analyses

Exploratory factor analysis (EFA) with principal axis factoring, promax rotation (k = 4), and extraction of factors based on eigenvalues higher than one was used to verify if there were more than one underlying construct within the measure. Prevalence of dental anxiety was determined for IDAF-4C+ and MDAS using the pre-defined cut-points for both scales. The U Mann–Whitney test was used to compare IDAF-4C+ and MDAS scores between males and females. Associations between IDAF-4C+ and MDAS scores were evaluated through Spearman’s correlation coefficients. Cronbach’s alpha was calculated to assess the reliability of the scales according to Nunnally parameters [48].

## 3. Results

Two hundred and thirty-two students were included in the study (response rate = 82.86%). Participants’ mean age was 23 years (SD ± 3.1 years), and most were female (67.2%, n = 156). Respondents’ characteristics are reported in Table 1.

From the exploratory factor analysis of the IDAF-4C+, it emerged that one factor explained 58.76% of the common variance (eigenvalues = 4.7).

Items in IDAF-4C+ reported factor loadings ranging from 0.65 to 0.88, which confirms the strong correlation of each item with a latent construct. IDAF-4C+ and MDAS and their subscales were correlated with coefficients ranging from 0.29 to 0.97 (Table 2).

Using MDAS cut points, 52.59% (n = 122) of participants had no dental fear, 43.10% (n = 100) had low/moderate dental fear, and 4.31% (n = 10) had high dental fear. Higher percentages of women showed low/moderate or high dental fear (women_low/moderate fear_ = 46.15%, men_low/moderate fear_ = 36.84%; women_high fear_ = 5.77%, men_high fear_ = 1.32%), though without any statistically significant difference (Table 3, part A). Using IDAF-4C+ cut-points, 92.67% (n = 215) of the participants had no dental fear, 3.88% (n = 9) had moderate fear, and 3.45% (n = 8) had high to extreme dental fear. Higher percentages of women reported moderate to high dental fear (4.49%, n = 7) and high to extreme dental fear (5.13%, n = 8). None of the men reported high to extreme dental fear (Table 3, part B).

No statistically significant difference emerged between males and females in IDAF-4C+ scores, although the women’s scores were higher than the men’s scores (Table 3, part A). Women reported significantly higher values for MDAS score (10.69 ± 4.60) and MDAS treatment-related anxiety (7.4 ± 3.24) (*p* < 0.01) (Table 4).

None of the participants showed dental phobia as previously defined (Table 5).

Five male participants answered “yes” to any of the IDAF-4P items. In contrast, women tended to report higher scores for IDAF-4S, although no statistically significant differences emerged (Table 6).

Cronbach’s alpha was 0.88 for IDAF-4C+ and 0.87 for MDAS.

## 4. Discussion

Dental anxiety is a complex situation characterized by an emotional response to stimuli or experiences associated with dental treatments [6], which results in heightened fear of dental procedures [10].

Patients with a high DA level can exacerbate discomfort associated with dental care, amplifying pain and discomfort before, during, and after the dental treatment [14]. Vice versa, some dental discomfort such as pain or sensitivity of disgust may be perceived as more disabling, thus inducing or heightening the patients’ DA.

Proper management of patients with DA may help clinicians reduce stress and negative consequences and possibly transform the vicious circle [19] into a virtuous circle from which the dentist–patient relationship is reinforced.

IDAF-4C+ may be a helpful tool for early detection of DA by dental professionals because it may assure better treatment outcomes [49]. From our findings, the Italian version of the IDAF-4C+ reported a high level of validity and reliability, thus representing an optimal measure for dental anxiety and fear among Italian-speaking adults.

As demonstrated by previous studies, dental anxiety and fear were more common among women than men in IDAF-4C+ and MDAS, although without statistically significant differences [9,50].

From the comparison between IDAF-4C+ and MDAS classifications, some relevant differences emerged. The number of participants with no dental fear was higher with IDAF-4C+ (92.67%) than with MDAS (52.59%). In contrast, IDAF-4C+ reported a lower percentage of participants with moderate fear (3.88%), while MDAS registered higher rates (43.10%) of participants with low/moderate dental fear. This relevant difference between the two measures could be due to the different cut-points used, which also emerged in Tolvanen et al., (2017), or could be the results of the different dimensions captured by the two scores [45]. MDAS mainly focuses on the emotional response to dental stimuli, while IDAF-4C+ is oriented to describe the whole cognitive impairment due to the dental setting [32].

Strong association was reported between MDAS and IDAF-4C+ scores (0.68, *p* < 0.001) and MDAS and physiological (0.58, *p* < 0.001) and emotional (0.53, *p* < 0.001) components. This finding is in line with previous studies [34,45], underlying once again the emotion-oriented characteristics of MDAS, correctly captured by the emotional and physiological IDAF-4C+ subscales.

Some dental procedures that could increase the level of DA inducing discomfort such as the duration of the procedures, pain overestimation [51], and the lack of insightful comprehension of the whole dental treatment [52] have been identified as possible risk factors for DA. Patients with a high level of anxiety are 2–9 times more likely to feel moderate or intensive intraoperative pain during canal treatment [51].

Having the Italian version of IDAF-4C+ allows Italian clinicians to improve the trust relationship with their patients and reduce the panic sensation proven by many adult patients before a specific surgical procedure such as implant surgery. Moreover, this validated tool will help standardize their research by applying a standardized method that measures dental anxiety at all levels of severity.

Despite our positive findings, some limitations emerged. Selection bias could have been determined given the convenience sample of university students (i.e., students attending medicine or dentistry). Hawthorne effect and bias due to self-reported assessment could also be present. Moreover, possible respondents with a higher level of DA could have decided not to participate in the survey.

## 5. Conclusions

The IDAF-4C+ Italian version is a valid and reliable tool to determine the presence and level of dental anxiety. It will help clinicians identify patients who suffer from dental anxiety, fear, or phobia in a more standardized way, evaluating dental anxiety, fear, and dental phobia with only one tool. In a country where more than 80% of patients have odontophobia, using a validated and cross-cultural adapted tool may encourage patients to start collaborative behaviors with their dentists.

For future research, it will be important to test the reliability and validity of this instrument in the specific categories of patients such as those who require more invasive dental surgery.

## Figures and Tables

**Table 1:** Respondents’ characteristics.

Number of Respondents	232
Gender	-
Female	156 (67.2%)
Male	76 (32.8%)
Age, mean (SD)	23.0 (3.1)

**Table 2:** Spearman’s correlation coefficient.

		IDAF-4C					MDAS	
		Total	Physiological	Behavior	Cognitive	Emotional	Total	AA
IDAF-4C	Physiological	0.89 *	-	-	-	-	-	-
	Behavioral	0.60 *	0.38 *	-	-	-	-	-
	Cognitive	060 *	0.38 *	0.29 *	-	-	-	-
	Emotional	0.70 *	0.58 *	0.44 *	0.48 *	-	-	-
MDAS	Total	0.68 *	0.58 *	0.47 *	0.48 *	0.53 *	-	-
	Anticipatory anxiety	0.68 *	0.66 *	0.46 *	0.42 *	0.59 *	0.75 *	-
	Treatment-related anxiety	0.61 *	0.50 *	0.42 *	0.46 *	0.48 *	0.97 *	0.58 *

AA: Anticipatory anxiety. * Statistical significance of correlation coefficient lower than 0.05 (*p* < 0.05).

**Table 3:** Prevalence of fear.

	Total (n = 232)	Female (n = 156)	Male (n = 76)	*p*
A. MDAS				
No Fear	122 (52.59%)	75 (48.08%)	47 (61.84%)	0.074
Low/Moderate Fear	100 (43.10%)	72 (46.15%)	28 (36.84%)	
High Fear	10 (4.31%)	9 (5.77%)	1 (1.32%)	
B. IDAF-4C				
No Fear	215 (92.67%)	141 (90.38%)	74 (97.37%)	0.099
Moderate Fear	9 (3.88%)	7 (4.49%)	2 (2.63%)	
High to Extreme Fear	8 (3.45%)	8 (5.13%)	0 (0.00%)	

**Table 4:** Descriptive statistics for the IDAF-4C and MDAS by gender.

		Total (n = 232)	Female (n = 156)	Male (n = 76)	*p*
IDAF-4C	Total	1.47 (± 0.67) 1.3 (1.0–1.6)	1.51 (± 0.77) 1.3 (1.0–1.6)	1.38 (± 0.42) 1.3 (1.0–1.6)	0.8378
	Physiological	1.81 (± 1.01) 1.5 (1.0–2.0)	1.89 (± 1.12) 1.5 (1.0–2.5)	1.66 (± 0.73) 1.5 (1.0–2.0)	0.4910
	Behavioral	1.37 (± 0.75) 1.0 (1.0–1.5)	1.38 (± 0.81) 1.0 (1.0–1.5)	1.35 (± 0.59) 1.0 (1.0–1.5)	0.6332
	Cognitive	1.33 (± 0.70) 1.0 (1.0–1.5)	1.35 (± 0.76) 1.0 (1.0–1.5)	1.28 (± 0.53) 1.0 (1.0–1.5)	0.9713
	Emotional	1.35 (± 0.76) 1.0 (1.0–1.5)	1.40 (± 0.87) 1.0 (1.0–1.5)	1.25 (± 0.47) 1.0 (1.0–1.5)	0.9141
MDAS	Total	10.09 (± 4.25) 9.0 (7.0–12.0)	10.69 (± 4.60) 10.0 (7.0–13.0)	8.86 (± 3.11) 8.0 (6.0–11.0)	0.0072
	Anticipatory anxiety	3.12 (± 1.60) 2.0 (2.0–4.0)	3.29 (± 1.80) 3.0 (2.0–4.0)	2.76 (± 0.99) 2.0 (2.0–3.5)	0.1247
	Treatment-related anxiety	6.97 (± 3.08) 6.0 (5.0–9.0)	7.4 (± 3.24) 7.0 (5.0–0.0)	6.09 (± 2.51) 6.0 (4.0–8.0)	0.0043

Values are reported as mean (SD) and median (25th–75th).

**Table 5:** IDAF-4P.

Item *	Total (n = 232)	Female (n = 156)	Male (n = 76)	*p*
P1	17 (7.33)	13 (8.33)	4 (5.26)	0.400
P2	23 (9.91)	18 (11.54)	5 (6.58)	0.235
P3	12 (5.17)	7 (4.49)	5 (6.58)	0.500
P4	7 (3.02)	3 (1.92)	4 (5.26)	0.163
P5	25 (10.78)	18 (11.54)	7 (9.21)	0.591

* English and Italian version of each item is reported in the Supplementary Materials.

**Table 6:** IDAF-4S.

Items *	Total (n = 232)	Female (n = 156)	Male (n = 76)	*p*
S1	2.51 (0.99)	2.59 (1.03)	2.34 (0.89)	0.1352
S2	1.51 (0.79)	1.56 (0.85)	1.42 (0.64)	0.4375
S3	1.66 (0.95)	1.69 (0.94)	1.58 (0.98)	0.1571
S4	1.45 (0.79)	1.50 (0.85)	1.36 (0.65)	0.2668
S5	1.59 (0.82)	1.62 (0.86)	1.51 (0.74)	0.5114
S6	1.71 (0.98)	1.74 (0.99)	1.63 (0.96)	0.29
S7	2.32 (1.19)	2.32 (1.16)	2.30 (1.27)	0.7076
S8	2.01 (1.19)	2.09 (1.23)	1.86 (1.10)	0.1464
S9	1.52 (0.87)	1.56 (0.90)	1.45 (0.82)	0.2994
S10	1.95 (1.16)	1.98 (1.18)	1.89 (1.11)	0.6889

* English and Italian version of each item is reported in the Supplementary Materials.

## Data Availability

Not applicable.

## Supplementary Materials

The following are available online at https://www.mdpi.com/article/10.3390/dj9120149/s1, Table S1: Italian IDAF-4C+ version.
