# Supplementary material for: Translation and Validation of Italian Version of Index of Dental Anxiety and Fear (IDAF-4C+): A Cross-Sectional Study
Source: Dent J (Basel). 2021 Dec 10;9(12):149. doi: 10.3390/dj9120149 (PMC8699893; doi:10.3390/dj9120149)
Supplement: Supplementary file 1 [file dentistry-09-00149-s001.zip › dentistry-1453323-supplementary.pdf]

**Table S1.** Italian IDAF-4C+ version.

| No. Item | English Version | Italian Version | Answers |
|---|---|---|---|
| IDAF-4C | | | |
| C1 | I feel anxious shorty before going to the dentist | Mi sento ansioso poco prima di andare dal dentista | In disaccordo to Fortemente d'accordo (5-point Likert Scale) |
| C2 | My heart beats faster when I go to the dentist | Il mio cuore batte più veloce quando devo andare dal dentista | In disaccordo to Fortemente d'accordo (5-point Likert Scale) |
| C3 | I delay making appointments to dentist | Ritardo nel prendere appuntamenti presso il dentista | In disaccordo to Fortemente d'accordo (5-point Likert Scale) |
| C4 | I generally avoid going to the dentist because I find the experience unpleasant or distressing | In genere evito di andare dal dentista perché trovo l'esperienza spiacevole o angosciante | In disaccordo to Fortemente d'accordo (5-point Likert Scale) |
| C5 | I think that something really bad would happen to me if I were to visit a dentist | Penso che potrebbe accadermi qualcosa di spiacevole se andassi dal dentista | In disaccordo to Fortemente d'accordo (5-point Likert Scale) |
| C6 | I often think about all the things that might go wrong prior to going to the dentist | Prima di andare dal dentista penso spesso a tutto ciò che può andare storto | In disaccordo to Fortemente d'accordo (5-point Likert Scale) |
| C7 | I get nervous or edgy about upcoming dental visits | Divento nervosa o irrequieto quando si approssimano le visite odontoiatriche | In disaccordo to Fortemente d'accordo (5-point Likert Scale) |
| C8 | I feel afraid or fearful when visiting the dentist | Mi sento impaurito o spaventato quando il dentista mi visita | In disaccordo to Fortemente d'accordo (5-point Likert Scale) |
| IDAF-4P | | | |
| P1 | Going to the dentist is actively avoided or else endured with intense fear or anxiety | Andare dal dentista è sempre evitato o sopportato con intensa paura ed ansia | SI/NO |
| P2 | My fear of going to the dentist has been present for at least 6 months | La mia paura di andare dal dentista è presente da almeno sei mesi | SI/NO |
| P3 | My fear, anxiety or avoidance of going to the dentist significantly affects my life in some way | La paura, l'ansia e l'evitare di andare dal dentista incidono significativamente sulla mia vita in molti modi (ad esempio ho dolore ai denti, evito di mangiare alcuni cibi, mi sento imbarazzato o impacciato per l'aspetto dei miei denti o della bocca) | SI/NO |
| P4 | I am afraid of going to the dentist because I am concerned I may have panic attack | Ho paura di andare dal dentista perché temo di avere un attacco di panico (paura improvvisa con sudorazione, cuore che batte forte, paura di morire o di perdere il controllo, dolore al petto) | SI/NO |
| P5 | I am afraid of going to the dentist because I am generally highly self-conscious or concerned about being watched or judged in social situations | Ho paura di andare dal dentista perché generalmente sono molto a disagio o preoccupato di essere osservato o giudicato in situazioni sociali | SI/NO |
| IDAF-S | | | |
| S1 | Painful or uncomfortable procedures | Procedure dolorose o scomode | Per nulla to Moltissimo (5-point Likert Scale) |

| | | | |
|---|---|---|---|
| S2 | Feeling embarrassed or ashamed | Sensazioni di imbarazzo o vergogna | Per nulla to Moltissimo (5-point Likert Scale) |
| S3 | Not in control of what is happening | Non avere il controllo di ciò che sta accadendo | Per nulla to Moltissimo (5-point Likert Scale) |
| S4 | Feeling sick, queasy or disgusted | Sensazione di malessere, nausea o disgusto | Per nulla to Moltissimo (5-point Likert Scale) |
| S5 | Numbness caused by the anaesthetic | Intorbidimento causato dall'anestestico | Per nulla to Moltissimo (5-point Likert Scale) |
| S6 | Not knowing what dentist is going to do | Non sapere cosa farà il dentista | Per nulla to Moltissimo (5-point Likert Scale) |
| S7 | The cost of dental treatment | Il costo del trattamento odontoiatrico | Per nulla to Moltissimo (5-point Likert Scale) |
| S8 | Needles or injections | Aghi o iniezioni | Per nulla to Moltissimo (5-point Likert Scale) |
| S9 | Gagging or choking | Ostruzione o soffocamento | Per nulla to Moltissimo (5-point Likert Scale) |
| S10 | Having an unsympathetic or unkind dentist | Avere un dentista antipatico o scortese | Per nulla to Moltissimo (5-point Likert Scale) |

English version is here presented as reported in Tolvanen et al. (2017) [45].
